# GlyT1 inhibition by ALX-5407 attenuates allograft rejection through suppression of Th1 cell differentiation

**DOI:** 10.3389/fimmu.2025.1644529

**Published:** 2025-09-23

**Authors:** Xiaohan Zhang, Weiqi Zhang, Jianghao Wei, Shuai Jin, Zhen Wang, Hui Wang, Gang Feng, Jie Zhao

**Affiliations:** ^1^ Research Institute of Transplant Medicine, School of Medicine, Tianjin First Central Hospital, Nankai University, Tianjin, China; ^2^ Department of Renal Transplantation, Tianjin First Central Hospital, Nankai University, Tianjin, China; ^3^ The First Central Clinical School, Tianjin Medical University, Tianjin, China

**Keywords:** solute carrier, GlyT1, ALX-5407, T-cell-mediated rejection, organ transplantation

## Abstract

**Objective:**

Transplant rejection driven by Th1 cell-mediated immune responses remains a critical challenge. This study aimed to investigate the role of glycine transporter 1 (GlyT1/SLC6A9) in Th1 differentiation and evaluate the therapeutic potential of its inhibitor, ALX-5407, in attenuating allograft rejection.

**Methods:**

RNA sequencing, flow cytometry, and qRT-PCR were employed to analyze GlyT1 expression in Th1-polarized CD4^+^T cells. ALX-5407 (0.5–500 nM) was tested *in vitro* under Th1-polarizing conditions. A murine skin allograft model (BALB/c to C57BL/6) was established to assess graft survival and immune responses. Combination therapy with rapamycin and ALX-5407 was evaluated through histopathology, immunofluorescence, and splenocyte profiling. Mechanistic insights were derived from RNA-seq, KEGG/GO enrichment, and Western blotting.

**Results:**

GlyT1 expression was significantly upregulated in Th1 cells and rejection cohorts. ALX-5407 suppressed Th1 differentiation, reducing IFN-γ^+^CD4^+^T cells proportions (p < 0.05) and activation markers (CD25, CD69), while inducing apoptosis via caspase-3 activation and BCL-2 downregulation. Although ALX-5407 monotherapy failed to prolong graft survival, combination with rapamycin synergistically enhanced efficacy (p = 0.018), reduced inflammatory infiltration, and attenuated splenic Th1 polarization. Mechanistically, ALX-5407 inhibited MAPK signaling but activated the PI3K-AKT-mTOR pathway, which rapamycin counteracted to amplify suppression.

**Conclusions:**

GlyT1 serves as a metabolic checkpoint in Th1 differentiation, and its inhibition by ALX-5407 attenuates allograft rejection through dual suppression of Th1 function and apoptosis induction. Synergy with rapamycin highlights a novel combinatorial strategy to mitigate rejection with reduced toxicity. These findings position GlyT1 targeting as a promising approach for clinical translation in transplantation immunotherapy.

## 1 Introduction

Transplant rejection remains a principal cause of graft loss, driven by dysregulated immune responses in which T helper 1 (Th1) cells play a central role (1, 2). Upon activation, T cells undergo metabolic reprogramming to meet bioenergetic demands, with enhanced glycolysis and mitochondrial oxidative phosphorylation emerging as critical regulatory nodes for effector function (3, 4). This metabolic plasticity not only sustains pro-inflammatory cytokine production but also establishes a potential therapeutic vulnerability. Targeting T cell metabolic pathways, such as glucose utilization or amino acid transport, may offer a promising strategy to attenuate pathological immune activation while preserving homeostatic immunity (5, 6).

Glycine functions as a pleiotropic metabolic hub with significant anti-inflammatory and anti-apoptotic properties (7). It acts as a one-carbon unit donor to drive cellular proliferation and epigenetic reprogramming via the one-carbon cycle, while serving as an essential substrate for glutathione (GSH) biosynthesis to scavenge reactive oxygen species (ROS), thereby alleviating oxidative stress and preserving cellular function. In the context of transplant rejection (8–10), the heightened metabolic activity of CD4^+^ Th1 cells renders them exquisitely sensitive to alterations in intracellular glycine concentrations (11, 12). Modulation of glycine availability selectively suppresses Th1 cell proliferation and effector functions, consequently attenuating Th1-dominated T cell-mediated allograft rejection and ultimately mitigating graft injury to prolong transplant survival. The solute carrier (SLC) transporter family, comprising over 400 membrane proteins, has recently been implicated in T cell metabolic adaptation (13, 14). Specific SLC members, including SLC7A5 (LAT1) and CD98, facilitate the uptake of essential nutrients such as leucine and glutamine, directly fueling mTORC1 signaling and epigenetic modifications during T cell differentiation (15, 16). Dysregulation of SLC-mediated metabolic rewiring correlates with aberrant CD4^+^T polarization in allograft rejection models, positioning this family as a novel immunomodulatory target. Elucidating the interplay between SLC transporters and T cell metabolism could uncover precision mechanisms to mitigate rejection without broad-spectrum immunosuppression (17).

SLC6A9(GlyT1), a member of the solute carrier 6 (SLC6) family, encodes the sodium/chloride-dependent glycine transporter 1 (18). GlyT1 transports glycine from the synaptic cleft back into neurons via active transport. ALX-5407, a potent and highly selective glycine transporter-1 inhibitor, effectively reduces glycine influx, leading to extracellular glycine accumulation. In the nervous system, this elevation of synaptic glycine concentration modulates neural plasticity and excitability, offering therapeutic potential for psychiatric disorders. This transporter primarily regulates glycine transmembrane flux and has recently been implicated in modulating mitochondrial glycine metabolism within cells (19). However, its specific mechanistic contributions to transplant rejection remain underexplored, warranting further investigation to elucidate its therapeutic potential in alloimmune regulation. based on the effect of GlyT1 inhibitors on reducing intracellular glycine levels and glycine’s critical role in CD4^+^T cell function, we propose the hypothesis that GlyT1 inhibition may modulate CD4^+^T cell activation states. Our study indicates that SLC6A9 plays a role in Th1 differentiation and is a potential target for reducing graft rejection.

## 2 Materials and methods

### 2.1 T cell isolation and polarization

Spleens were aseptically harvested from euthanized male C57BL/6 mice (male, 6-8w) and mechanically dissociated into single-cell suspensions. CD4^+^T cells were isolated using magnetic bead-based negative selection kits (STEMCELL) and cultured in anti-CD3ϵ pre-coated 96-well plates (5μg/mL, BioLegend) under Th1-polarizing conditions: RPMI 1640 medium supplemented with recombinant IL-2 (100 IU/mL, PeproTech), IL-12p70 (10 ng/mL, PeproTech), anti-IL-4 (10μg/mL, BioLegend), and anti-CD28 (2μg/mL, BioLegend). ALX-5407 (MCE HY-10711A) was administered at 0.5–500 nM concentrations, with vehicle-treated cells serving as controls. Cells were harvested after 72-hour stimulation for flow cytometry or RNA sequencing.

### 2.2 Mouse skin transplantation model

Full-thickness tail skin grafts (1×1 cm) from donor BALB/c mice (male, 6-8w) were transplanted onto the dorsal region of anesthetized C57BL/6 recipients (male, 6-8w) using 6–0 Prolene sutures. Postoperatively, mice received daily intraperitoneal injections of ALX-5407 (100 mg/kg), rapamycin (50 mg/kg), or vehicle (n=5/group). Graft viability was monitored until Day 7, with >90% necrosis defined as rejection. Surviving grafts and secondary lymphoid organs were collected for histopathological and immunological analyses.

### 2.3 Mouse heart transplantation model

BALB/c mice (male, 6-8w) were anesthetized by inhalation and systemically heparinized via intraperitoneal injection. The thoracic cavity of the mouse was then opened. After the heart stopped beating, the superior vena cava and pulmonary veins of the mouse heart were ligated, and the aorta and pulmonary artery were disconnected. The donor heart was harvested and stored in ice-cold saline. C57BL/6 mice (male, 6-8w) were anesthetized by inhalation, and the skin on the right neck was incised to locate the right internal jugular vein and the right internal carotid artery. The right internal jugular vein and the right internal carotid artery were ligated, disconnected, and cannulated. The donor heart aorta was then sleeved onto the recipient’s internal carotid artery end, and the donor heart pulmonary artery was sleeved onto the recipient’s internal jugular vein end. After fixing and ligating the interfaces, blood flow was restored, and the heart filled and restarted beating. After the operation, the recipient mice were anesthetized by inhalation daily, and the beating of the donor heart was assessed by manual palpation to determine the heart beating situation, and a graft survival curve was plotted.

### 2.4 Flow cytometry

Single-cell suspensions from *in vitro* cultures or recipient spleens were stained with fluorochrome-conjugated antibodies against surface markers (Live/Dead, CD4, CD8α, CD25, CD44, CD69, NK1.1, CD19, CD11b, Ly6G, Ly6C, CD11c, F4/80), followed by intracellular fixation/permeabilization for IFN-γ, IL-17A, and Foxp3 detection. For cytokine profiling, cells were stimulated with PMA/ionomycin (1 μg/mL each, Invitrogen™) in the presence of brefeldin A (10 μg/mL, Invitrogen™) for 6 hours prior to staining. Data acquisition was performed on a BD LSR Fortessa X-20 cytometer and analyzed using FlowJo v10.8.

### 2.5 qRT-PCR

Total RNA was extracted from The CD4^+^T cells sorted from the spleen and subsequently cultured *in vitro* for 3 days using TRIzol reagent (Invitrogen) and reverse-transcribed into cDNA with PrimeScript RT Master Mix (TransGen). qPCR was performed on a LightCycler^®^ 96 system (Roche) using 2X SYBR Green Premix (TransGen) in 10 μL reactions. Primer pairs were designed via NCBI Primer-BLAST and validated by melting curve analysis. Relative gene expression was calculated using the 2^(-ΔΔCt) method with GAPDH as endogenous control. All reactions were performed in triplicate.

### 2.6 H&E staining and immunofluorescence

Transplanted skin grafts and transplanted heart grafts were fixed in 4% paraformaldehyde, paraffin-embedded, and sectioned at 5 μm thickness. Hematoxylin and eosin (H&E) staining was performed using standard protocols (Servicebio) for histopathological evaluation. For CD4 immunofluorescence, antigen retrieval was performed with citrate buffer (pH 6.0, 95°C, 15 min). Sections were blocked with 10% goat serum (Beyotime) and incubated with anti-CD4 primary antibody (1:100, Invitrogen) at 4°C overnight, followed by Alexa Fluor 488-conjugated secondary antibody (1:500, Invitrogen) for 1 h at room temperature. Nuclei were counterstained with DAPI (Servicebio). Images were captured using a Olympus upright fluorescence microscope. Negative controls omitted primary antibodies.

### 2.7 CFSE proliferation assay and *in vivo* CTV proliferation assay

The CD4^+^T cells sorted from the spleen were labeled with 5 μM CFSE (Invitrogen) in PBS at 37°C for 10 min, followed by quenching with complete RPMI-1640 medium. Cells were seeded in 96-well plates and treated with ALX-5407 at concentrations of 0.5, 5, 50, or 500 nM, while control groups received vehicle (0.1% DMSO). After 72 h incubation (37°C, 5% CO_2_), cells were harvested and analyzed by flow cytometry (BD LSR Fortessa 20X). Proliferation indices were calculated using FlowJo v10.8 software based on CFSE dilution.

The CD4^+^ T cells sorted from the spleens of C57BL/6 mice were labeled with CellTrace™ Violet (CTV) (20) and activated *in vitro* for 1 day using anti-mouse CD3 (5 ng/ml) and anti-mouse CD28 (2 ng/ml). They were then intravenously infused into C57BL/6 mice, which were randomly divided into an ALX-5407 group and a control group. According to the group, mice received intraperitoneal injections of ALX-5407 (50 mg/kg, once daily)(n=5). Three days later, spleens were collected from the mice for flow cytometry analysis to compare the proliferation of CD4^+^ CTV^+^ cells between the two groups by flow cytometry (BD LSR Fortessa 20X). Proliferation indices were calculated using FlowJo v10.8 software based on CTV dilution.

### 2.8 RNA sequencing

The CD4^+^T cells sorted from the spleen were polarized into Th0 and Th1 subsets under standard conditions for 3 days. For ALX-5407 treatment, cells were exposed to 500 nM ALX-5407 or vehicle (0.1% DMSO) for 24 h. Total RNA was extracted using TRIzol (Invitrogen), followed by ribosomal RNA depletion and library preparation with NEBNext Ultra II RNA Library Prep Kit (NEB). Differentially Expressed Genes (DEGs) were identified using DESeq2 (|log2FC| >1, adj. p <0.05). Gene Ontology (GO) and KEGG pathway analyses were conducted via clusterProfiler (v4.0).

### 2.9 Western blotting

The CD4^+^T cells sorted from the spleen and subsequently cultured *in vitro* for 3 days were lysed in RIPA buffer (Beyotime) containing protease inhibitors (Beyotime). Protein concentrations were quantified via BCA assay (Thermo Fisher). Equal amounts (20 μg/lane) were separated by 10% SDS-PAGE gel electrophoresis and transferred onto PVDF membranes (Millipore). After blocking with 5% non-fat milk for 1 h, membranes were incubated overnight at 4°C with primary antibodies as loading control. HRP-conjugated secondary antibodies were applied for 1 h at RT. Signals were detected using ECL chemiluminescence and quantified by ImageJ v1.53.

### 2.10 Statistical analysis

Data were analyzed using GraphPad Prism v10.0 and expressed as mean ± SD. Multiple group comparisons employed two-way ANOVA with Bonferroni *post hoc* correction. Pairwise comparisons between specific groups used unpaired Student’s t-test. Skin graft survival rates were analyzed by Kaplan-Meier method with log-rank test for significance. p < 0.05 was considered to be statistically significant.

## 3 Results

### 3.1 SLC6A9 (GlyT1) is highly expressed in Th1 cells

**Figure 1:**
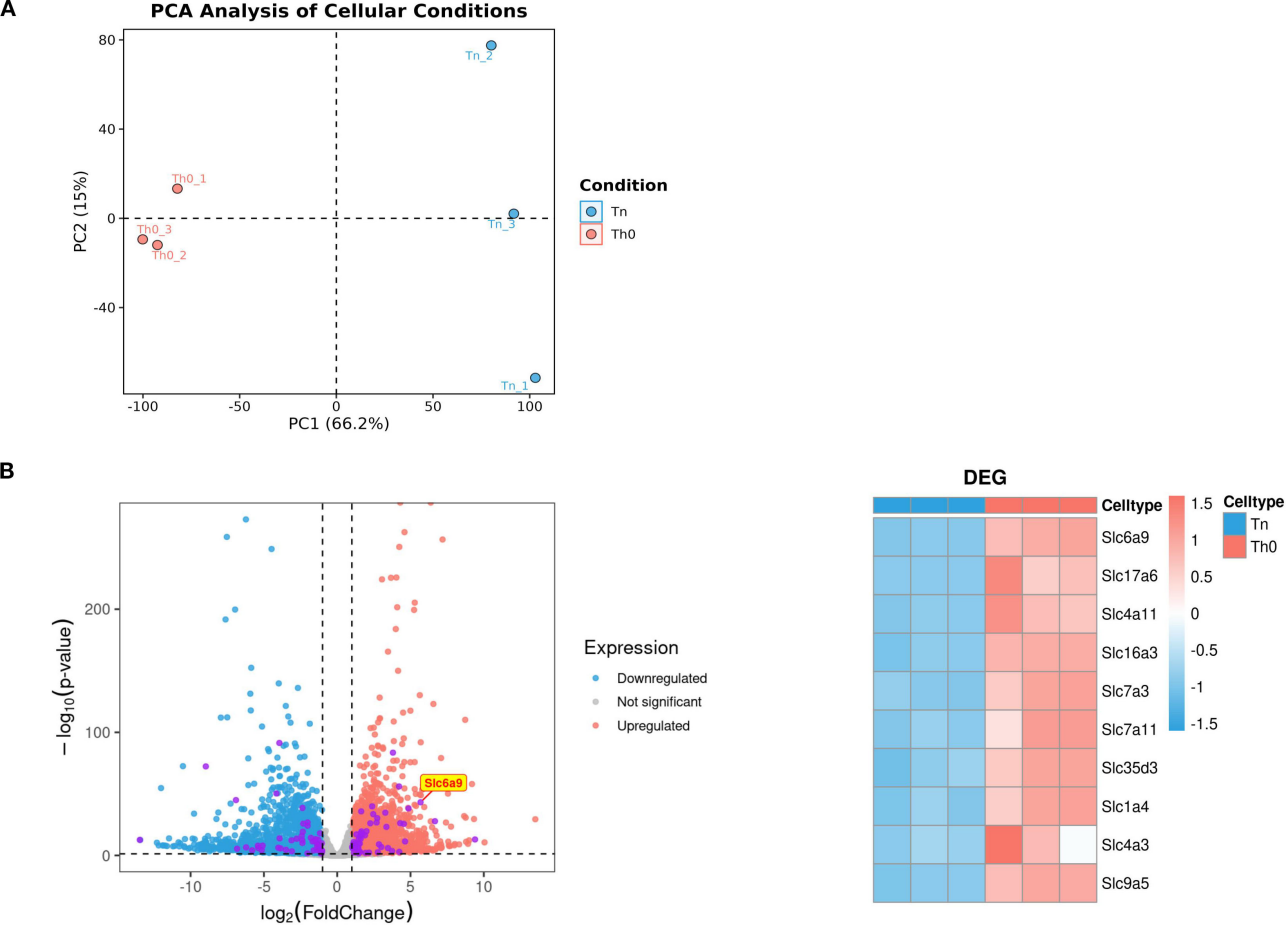
GlyT1 is highly expressed in Th0 cells. CD4^+^T cells were isolated from the spleens of C57BL/6 mice using magnetic bead separation and cultured under Naive and Th0 conditions for three days. After three days, RNA was extracted for RNA sequencing. Differential expression analysis was performed between the two groups **(A)**, with subsequent focus on the top 10 most differentially expressed genes from the solute carrier (SLC) family (Purple dot) for detailed characterization **(B)**. (n = 3).

To investigate differences between Th0 and Th1 cells, CD4^+^T cells isolated from spleens of male C57BL/6 mice via magnetic bead sorting were cultured under Th0 or Th1 conditions for 3 days. RNA sequencing revealed distinct transcriptomic profiles between Th0 and Th1 cells (**Figure 1A**). Solute carrier (SLC) family proteins, as primary transporters of intracellular substances, profoundly influence cellular metabolic homeostasis through expression changes. Therefore, we prioritized analysis of differential SLC protein expression between the two experimental groups. Among differentially expressed genes, SLC6A9 (GlyT1), a cell-surface glycine transporter critical for maintaining intracellular/extracellular glycine homeostasis, exhibited significantly higher expression in Th1 cells compared to Th0 cells (p < 0.05, **Figure 1B**). These findings suggest that GlyT1-mediated glycine regulation may influence Th1 proliferation and differentiation.

### 3.2 ALX-5407 does not affect the proliferation of CD4^+^ T cells *in vivo* and *in vitro* but impacts the differentiation and function of CD4^+^ Th1 cells

**Figure 2:**
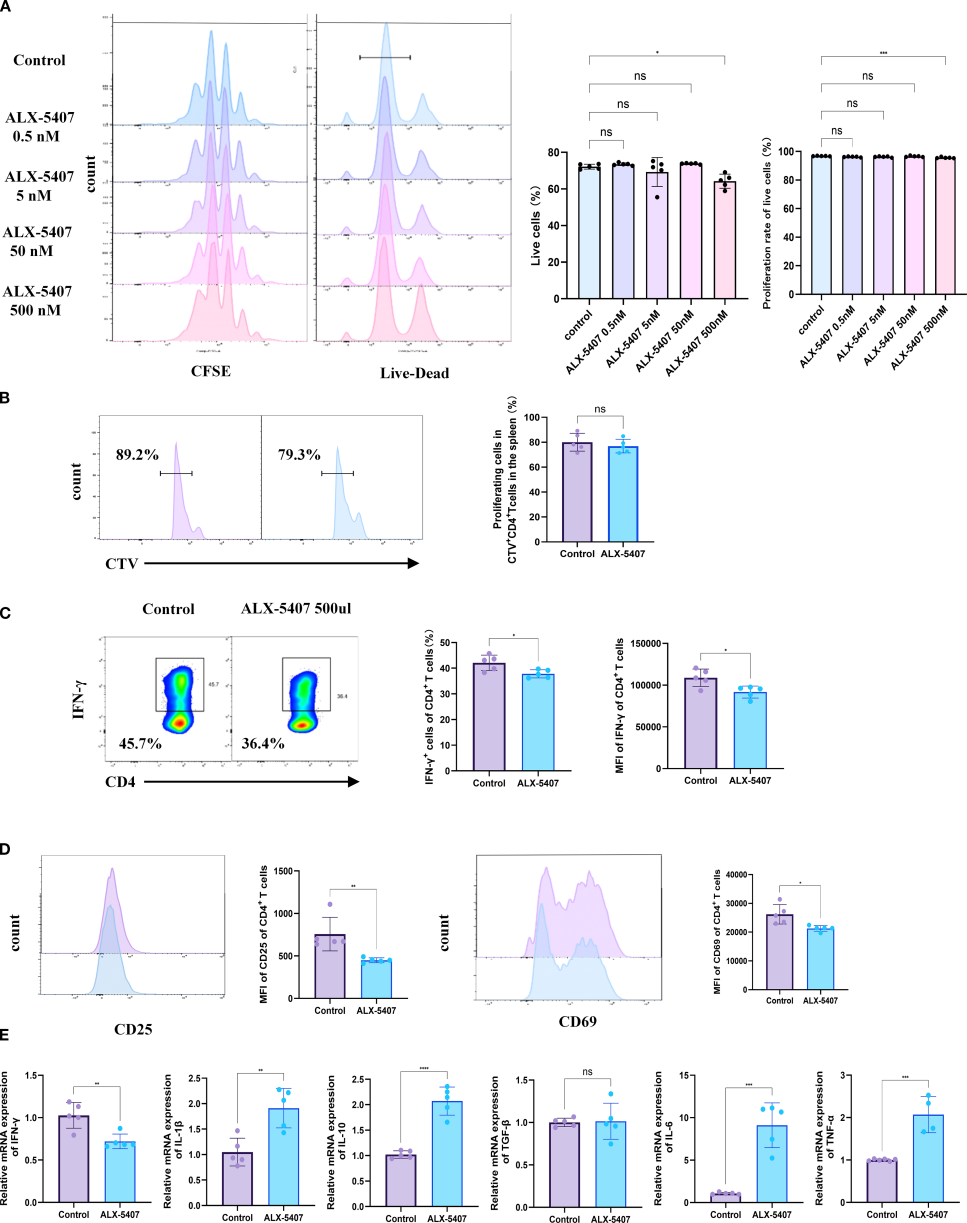
ALX-5407 effectively inhibits Th1 cell differentiation but has limited effects on proliferation. Magnetic bead separation was used to isolate CD4^+^ T cells from the spleens of C57BL/6 mice. These cells were then treated with varying concentrations of ALX-5407 (0.5 nM, 5 nM, 50 nM, and 500 nM) in culture medium. The CD4^+^ T cells were activated and cultured under Th1 conditions for three days. A portion of the cells were labeled with CFSE and cultured for three days, with the control group serving as a reference. After three days, cells were collected for flow cytometry analysis to assess survival rates **(A)** and CFSE staining to evaluate proliferation **(A)**. CD4^+^ T cells sorted from the spleens of C57BL/6 mice were labeled with Cell Trace Violet (CTV) and activated *in vitro* for 1 day using anti-mouse CD3(5 ng/ml) and anti-mouse CD28 (2 ng/ml). They were then intravenously infused into C57BL/6 mice, which were randomly divided into an ALX-5407 group and a control group. According to the group, mice received intraperitoneal injections of ALX-5407 (50 mg/kg, once daily) (n=5). Three days later, spleens were collected from the mice for flow cytometry analysis to compare the proliferation of CD4^+^CTV^+^T cells between the two groups **(B)**. Flow cytometry was also employed to detect the proportion of CD4^+^ IFN-γ^+^T cells **(C)** and the mean fluorescence intensity(MFI)of CD25 and CD69 **(D)**. qPCR was performed to examine the mRNA expression of various cytokines in the cells **(E)**. Statistical differences between groups were analyzed using the ANOVA test and T-test. (n =5; *p < 0.05, **p < 0.01, ***p < 0.001, ****p < 0.0001 ns:no significance).

To investigate whether GlyT1 could influence CD4^+^ T cell proliferation and differentiation, we utilized ALX-5407, a specific inhibitor of GlyT1. According to literature, ALX-5407 selectively inhibits GlyT1 function by reducing glycine transmembrane flux and is currently under experimental investigation for treating neuropsychiatric disorders, with a recommended concentration of 5 nM for neuronal cells. To determine the impact of ALX-5407 on Th1 differentiation and identify effective doses, we treated CFSE-labeled CD4^+^T cells with a concentration gradient (0.5 nM, 5 nM, 50 nM, 500 nM) under Th1-polarizing conditions for 3 days. Flow cytometry results after 3 days showed that ALX-5407 at various concentrations had minimal effects on Th1 cell proliferation (**Figure 2A**). However, cell viability began to decline at 500 nM, prompting us to select this concentration for subsequent experiments. To further investigate the impact of ALX-5407 on CD4^+^T cells proliferation *in vivo*, we isolated CD4^+^T cells from the spleens of C57BL/6 mice, labeled them with CTV (20), and activated them *in vitro*. These cells were then injected into C57BL/6 mice. Flow cytometry analysis of splenocytes 3 days post-injection revealed that the proliferation of CD4^+^CTV^+^cells in the ALX-5407 treatment group appeared moderately reduced compared to the control group. However, this result showed no statistical significance, suggesting that, consistent with *in vitro* results, ALX-5407 exerts minimal impact on CD4^+^T cell proliferation *in vivo* (**Figure 2B**).

However, Flow cytometry analysis revealed that the proportion of CD4^+^IFN-γ^+^ T cells in the ALX-5407-treated group was significantly lower than in the control group, with IFN-γ MFI also markedly reduced (**Figure 2C**). Concurrently, MFI values of CD25 and CD69 in the ALX-5407 group decreased, indicating reduced T cell activation (**Figure 2D**). Further analysis of cytokine mRNA expression levels demonstrated consistent results: *Ifng* expression was significantly downregulated, while *Il1β* and *Il10* were upregulated, with no change in *Tgfb1* (**Figure 2E**). These results suggest that ALX-5407, a GlyT1 inhibitor, significantly suppresses Th1 differentiation and impairs Th1 cell activation and function.

### 3.3 ALX-5407 synergizes with rapamycin to prolong allograft survival

**Figure 3:**
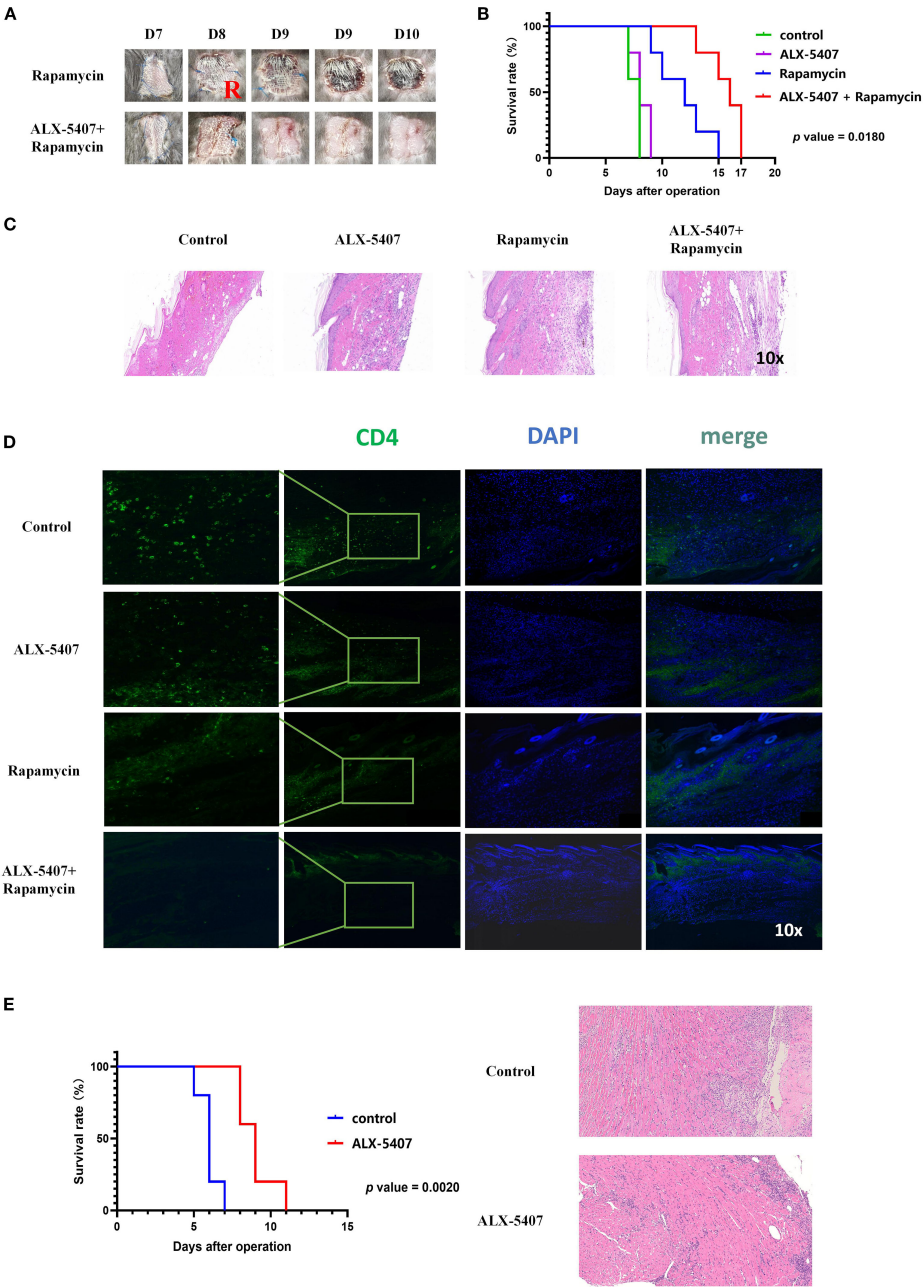
ALX-5407 combined with rapamycin effectively prolonged skin allograft survival in mice. BALB/C mouse skin was transplanted onto the backs of C57BL/6 mice, which were randomly divided into two groups: the rapamycin group and the ALX-5407+ rapamycin group. Starting from the day of surgery, the mice in each group were administered daily intraperitoneal injections of rapamycin (50 mg/kg) alone or rapamycin (50mg/kg) combined with ALX-5407 (100mg/kg). Grafts were photographed daily to assess rejection **(A)**, and survival curves were plotted **(B)**. On postoperative day 7, five mice from each group were randomly selected, euthanized, and their transplanted skin was collected for histological examination using HE staining **(C)** to evaluate rejection and immunofluorescence staining **(D)** to assess the infiltration of CD4^+^T cells in the grafts and the mean fluorescence intensity of CD4 expression within each group was analyzed using ImageJ (n=5). Using the cuff technique, cardiac allografts from BALB/c donor mice were heterotopically transplanted into the cervical region of C57BL/6 recipient mice, which were then randomly allocated into four groups: control (no treatment), ALX-5407 (100 mg/kg daily), rapamycin (50 mg/kg daily), and ALX-5407 plus rapamycin (100 mg/kg and 50 mg/kg daily, respectively). Beginning on the day of surgery, mice received daily intraperitoneal injections of the designated treatments, and graft rejection was assessed through daily photographic documentation, and survival curves were plotted **(E)**. On postoperative day 7, five mice from each group were randomly selected, euthanized, and their transplanted heart was collected for histological examination using HE staining **(E)** to evaluate rejection. Statistical differences between groups were analyzed using the ANOVA test and T-test. (n =5).

To further validate our findings, we established a BALB/c (male, 6–8 weeks) to C57BL/6 (male, 6–8 weeks) murine skin allograft model. Starting from the day of surgery, four groups received daily intraperitoneal injections: control, ALX-5407 alone, rapamycin alone, and ALX-5407 combined with rapamycin. Graft survival analysis demonstrated that ALX-5407 monotherapy failed to prolong graft survival, while the combination treatment significantly extended survival compared to both single-agent groups (p=0.018, **Figure 3A-B**). Histological evaluation on day 7 post-transplantation revealed better-preserved graft architecture with reduced inflammatory infiltration in the combination group (**Figure 3C**). Immunofluorescence analysis further confirmed significantly fewer CD4^+^T cells infiltrating the grafts in the combination group compared to other treatments (**Figure 3D** and **Supplementary Figure 2**). These results indicate that ALX-5407 synergizes with rapamycin to exert significantly enhanced protective effects on allografts.

Based on the above skin transplantation results, to further clarify the impact of ALX-5407 on solid organ transplantation, we established a mouse allogeneic heart transplantation model for validation. Experimental data demonstrated that the survival time of the transplanted heart in the ALX-5407 treatment group was significantly prolonged compared with the control group, with a statistically significant difference (**Figure 3E**, p=0.0020). Pathological analysis of the transplanted heart tissue collected on post-transplantation day 7 revealed that, compared with the control group, the structural integrity of the transplanted heart in the ALX-5407 treatment group was significantly improved, and the degree of inflammatory cell infiltration was notably reduced (**Figure 3E**).

### 3.4 ALX-5407 attenuates Th1-driven rejection by modulating splenic immune responses

**Figure 4:**
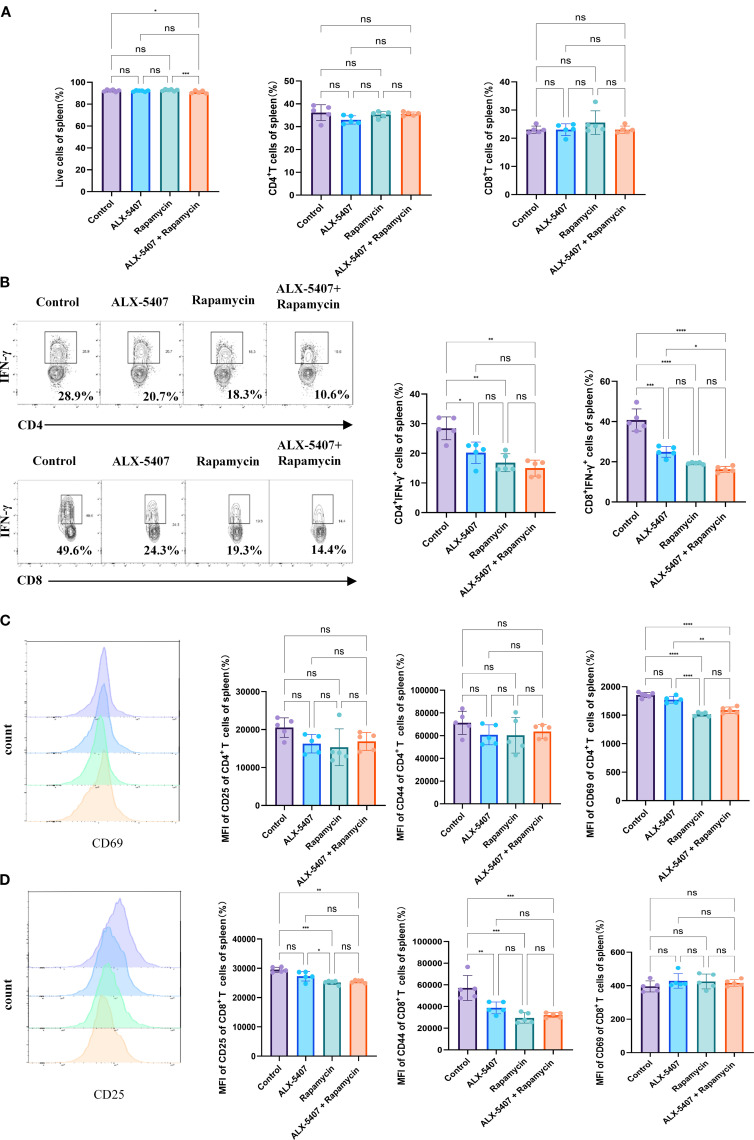
ALX-5407 improves graft survival by inhibiting the proportion and function of Th1 cells in skin-transplanted mice. Skin from BALB/C mice was transplanted onto the backs of C57BL/6 mice, which were randomly divided into four groups: a control group, an ALX-5407 group, a Rapamycin group, and an ALX-5407 + Rapamycin group. Daily intraperitoneal injections of the respective drugs were administered post-surgery according to the group assignments. On day 7 after transplantation, the mice were euthanized, and their spleens and peripheral blood were collected for flow cytometry analysis. The proportions of CD4^+^T cells and CD8^+^T cells in the spleen **(A)** and the proportions of CD4^+^ IFN-γ^+^ T cells and CD8^+^ IFN-γ^+^ T cells, along with their mean fluorescence intensity **(B)**, were detected. Additionally, the expression levels of CD25, CD44, and CD69 were measured to assess T cell functionality **(C, D)**. Differences between groups were analyzed using ANOVA test. (n =5; *p < 0.05, **p < 0.01, ***p < 0.001, ****p < 0.0001 ns:no significance).

To investigate the mechanisms underlying the protective effects of ALX-5407 combined with rapamycin on allografts, we performed flow cytometry analysis on splenocytes from skin-grafted mice at postoperative day 7 (gating strategy shown in **Supplementary Figure 1**). No significant differences were observed among groups in splenocyte viability, CD4^+^/CD8^+^ T cell ratios, or CD4^+^/CD8^+^ T cell proportions (**Figure 4A**). Notably, the proportion of CD4^+^IFN-γ^+^ T cells was reduced in all treatment groups compared to controls, with the most pronounced reduction in the combination group (**Figure 4B**). A parallel reduction was observed in CD8^+^IFN-γ^+^ T cell frequencies. Furthermore, combination therapy significantly reduced activation marker expression (CD25, CD44, CD69 MFI) in both CD4^+^ and CD8^+^ T cells (**Figures 4C, D**). These findings demonstrate that ALX-5407 and rapamycin synergistically suppress Th1 polarization while attenuating T cell activation.

To determine whether ALX-5407 impacts other T cell subsets, we analyzed regulatory T cells (Tregs) and Th17 cells, which showed no significant intergroup differences (**Supplementary Figure 3**). Analysis of broader immune populations, including B cells, NK cells, macrophages, dendritic cells, and myeloid-derived suppressor cells, revealed no treatment-associated alterations. Collectively, these data indicate that the anti-rejection efficacy of ALX-5407 combined with rapamycin primarily stems from coordinated inhibition of Th1 differentiation and functional activity.

### 3.5 ALX-5407 suppresses Th1 cell differentiation via apoptosis induction

**Figure 5:**
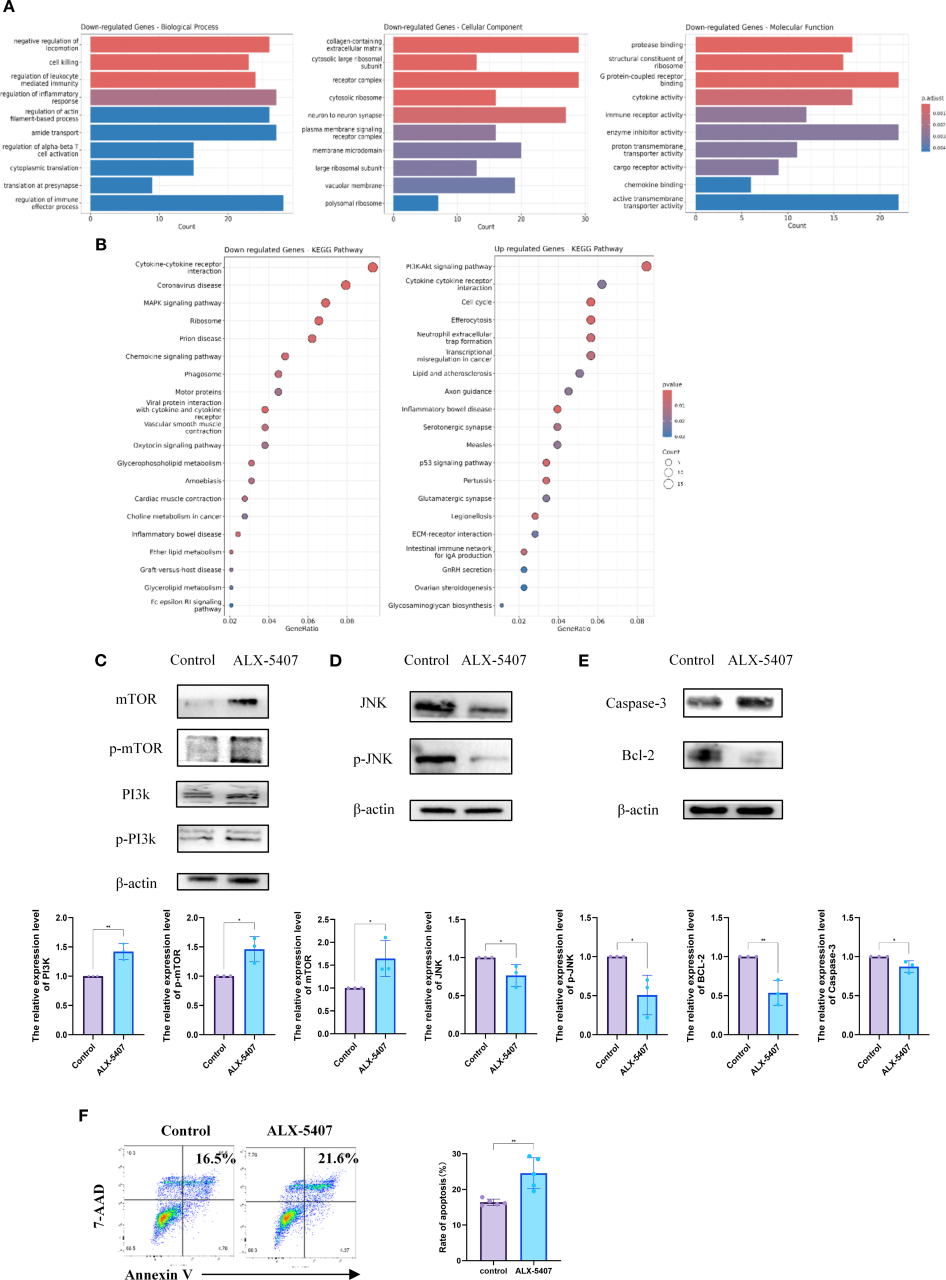
ALX-5407 inhibits Th1 cell differentiation by downregulating the MAPK pathway and increasing cell apoptosis. Magnetic bead separation was used to isolate CD4^+^T cells from the spleens of C57BL/6 mice, which were then treated with ALX-5407 (500 nM) in culture medium. The CD4^+^T cells were activated and cultured under Th1 conditions for three days, with the control group serving as a reference. After three days, RNA was extracted from the cells and subjected to RNA-sequencing. Functional analysis was performed using the GO database **(A)** and KEGG pathway enrichment **(B)** to identify differentially expressed genes and enriched pathways. Protein was extracted from the cells for Western blot analysis to detect differences in protein expression and the relative expression levels of the protein were compared between the two groups (n=3). **(C–E)**. Additionally, flow cytometry was used to detect differences in cell apoptosis between groups (n=5) **(F)**. Statistical differences between groups were analyzed using the T-test. (*p < 0.05, **p < 0.01 ns:no significance).

To investigate the mechanism by which ALX-5407 inhibits Th1 differentiation, we performed RNA sequencing (RNA-seq) on ALX-5407-treated Th1 cells and conducted KEGG/GO enrichment analyses compared to untreated controls (**Figures 5A, B**). The results revealed significant upregulation of the PI3K-AKT-mTOR pathway and downregulation of the MAPK pathway in ALX-5407-treated cells, which were further validated by Western blot analysis (**Figures 5C–E**). Given the critical roles of PI3K-AKT-mTOR and MAPK pathways in apoptosis regulation, we assessed apoptotic activity in ALX-5407-treated cells. Apoptosis was significantly increased in the ALX-5407 group (**Figure 5F**), with Western blot confirming elevated levels of cleaved caspase-3 and reduced expression of the anti-apoptotic protein BCL-2 (**Figure 5D**) (21, 22). These findings suggest that ALX-5407 reduces the Th1 cell population by inducing cellular apoptosis.

Notably, PI3K-AKT-mTOR pathway activation promotes T cell proliferation and differentiation, potentially explaining the limited efficacy of ALX-5407 monotherapy. Combining ALX-5407 with the mTOR inhibitor rapamycin partially counteracted mTOR pathway activation induced by ALX-5407, resulting in a synergistic enhancement of Th1 suppression (1 + 1>2 effect).

## 4 Discussion

The solute carrier (SLC) superfamily comprises over 65 subfamilies (SLC1-SLC65) and 400+ members with conserved structural features, functioning as transmembrane transporters for ions, metabolites, and pharmaceuticals (23–25). CD4^+^ Th1 cells play a pivotal role in transplant rejection, undergoing metabolic reprogramming with enhanced metabolic activity upon activation and Th1 differentiation (26). SLC family proteins critically participate in these processes and represent promising therapeutic targets for T cell modulation.

GlyT1(SLC6A9), predominantly expressed on neuronal cells, regulates glycine concentrations in neuronal somata and synaptic clefts, and has been explored for neuropsychiatric therapeutics (27–30). Its role in CD4^+^T cell biology remains unclear. Our RNA-seq analysis revealed significant upregulation of GlyT1 during Th0-to-Th1 differentiation, showing the most pronounced expression changes among screened SLC members, thus identifying GlyT1 as our research focus.

Glycine functions as a multifunctional metabolic hub, where its intracellular depletion reduces one-carbon unit availability, thereby limiting S-adenosylmethionine (SAM) biosynthesis (31). This deficiency exerts dual consequences: (1) Diminished provision of C4/C5/N7 atoms essential for purine ring formation impairs thymidylate synthesis, depleting DNA replication substrates and ultimately causing cell cycle arrest with reduced proliferative capacity (32); (2) Compromised methyl donor supply directly disrupts lineage-specific transcriptional programs (attenuated histone modification at the Tbx21 locus), altering epigenetic regulation in CD4^+^T cells (33–35). Concurrently, glycine deficiency inhibits glutathione (GSH) biosynthesis, promoting mitochondrial reactive oxygen species (ROS) accumulation in CD4^+^T cells (36). The inherently low antioxidant capacity in differentiating cells subsequently triggers apoptosis. Experimental GlyT1 inhibition recapitulated this pathophysiology, reducing intracellular glycine and resulting in: elevated caspase-3, suppressed BCL-2, increased CD4^+^T cell apoptosis, and impaired Th1 differentiation. These findings confirm glycine’s critical role in CD4^+^ T cell metabolism and demonstrate that targeting its metabolic flux induces functional reprogramming. Notably, in transplantation models, GlyT1 inhibition significantly reduced IFN-γ production in splenic CD8^+^ T cells-a pivotal cytotoxic T lymphocyte (CTL) effector molecule-suggesting parallel mechanisms may attenuate allograft damage, indicative of ALX-5407’s broader immunomodulatory potential.

However, unlike previous reports, CD4^+^T cell proliferation remained unaffected. RNA-seq analysis revealed compensatory PI3K-mTOR hyperactivation driving enhanced glycolysis to sustain proliferation. However, this metabolic adaptation simultaneously increased ROS generation and suppressed GSH synthesis, ultimately activating a ROS-BCL-2 axis that establishes the paradoxical “sustained proliferation with accelerated apoptosis” phenotype through failed homeostatic adaptation.

Current conventional immunosuppressive regimens, including glucocorticoids, calcineurin inhibitors (CNIs), mycophenolate mofetil (MMF), and mTOR inhibitors, exhibit notable cytotoxicity and potent immunosuppressive effects that increase risks of malignancies, infections, and systemic toxicities (37–39). This underscores the need for adjuvant immunosuppressive agents with reduced cytotoxicity that can potentiate conventional therapies while maintaining anti-rejection efficacy. Our findings demonstrate that ALX-5407 monotherapy in murine skin transplantation models showed marginal extension of graft survival compared to controls, though without statistical significance (p>0.05). Given ALX-5407’s demonstrated inhibition of Th1 differentiation *in vitro*, we investigated its combinatorial use with rapamycin. The combined regimen significantly prolonged allograft survival compared to either agent alone (p=0.018), with concomitant suppression of Th1 cell proportions in splenic tissues and CD4^+^ T cell infiltration in grafts. This synergistic effect suggests mutual potentiation between ALX-5407 and rapamycin. Mechanistic analysis revealed that ALX-5407 monotherapy induced paradoxical upregulation of the PI3K-Akt-mTOR pathway, which is a critical promoter of CD4^+^T cell proliferation/differentiation, potentially explaining its limited standalone efficacy. Crucially, rapamycin coadministration suppressed this feedback activation of mTOR, providing a plausible mechanism for the enhanced anti-rejection effects. This therapeutic strategy establishes a novel paradigm for developing adjuvant immunosuppressants through pathway complementarity (40–42).

Our preliminary evidence suggests ALX-5407 may suppress Th1 differentiation partially through apoptosis induction. Future studies will elucidate its precise molecular targets and optimize its anti-rejection efficacy through mechanism-based engineering.

## 5 Conclusion

GlyT1 serves as a metabolic checkpoint during Th1 differentiation. Targeting GlyT1 with ALX-5407 reduces Th1 polarization and prolongs allograft survival, likely through apoptosis induction. These findings position ALX-5407 as a novel immunomodulatory agent warranting further development in combination therapies for transplant rejection.

## Data Availability

Bulk RNA-seq data used in the generation of **Figures 1** and **5** are available through Gene Expression Omnibus (https://www.ncbi.nlm.nih.gov/geo/) under accession GEO: GSE307193. The original contributions presented in the study are included in the article/**Supplementary Material**. Further inquiries can be directed to the corresponding authors.

## Supplementary material

Supplementary Figure 1 Flow cytometry gating logic diagram showing the detection of cellular proportions *in vitro* and *in vivo* in this study.

Supplementary Figure 2 Enlarged images of CD4 immunofluorescence staining in mouse transplanted skin graft sections in **Figure 3D**, and the comparison of CD4 fluorescence expression levels among different groups.

Supplementary Figure 3 ALX-5407 does not statistically significantly affect other cell populations in the spleen of skin-transplanted mice. BALB/C mouse skin was transplanted onto the backs of C57BL/6 mice, which were then randomly divided into four groups: control, ALX-5407, rapamycin, and ALX-5407 + rapamycin. Following the surgery, each group received daily intraperitoneal injections of their respective treatments. Seven days post-surgery, the mice were euthanized, and their spleens and peripheral blood were collected for flow cytometry analysis. The proportions of various cell populations across the groups were compared using ANOVA or T-tests. (n =5;*p < 0.05, ** p < 0.01, *** p < 0.001, **** p < 0.0001).
