# Supplementary figures and images for: GlyT1 inhibition by ALX-5407 attenuates allograft rejection through suppression of Th1 cell differentiation
Source: Front Immunol. 2025 Sep 23;16:1644529. doi: 10.3389/fimmu.2025.1644529 (PMC12500449; doi:10.3389/fimmu.2025.1644529)

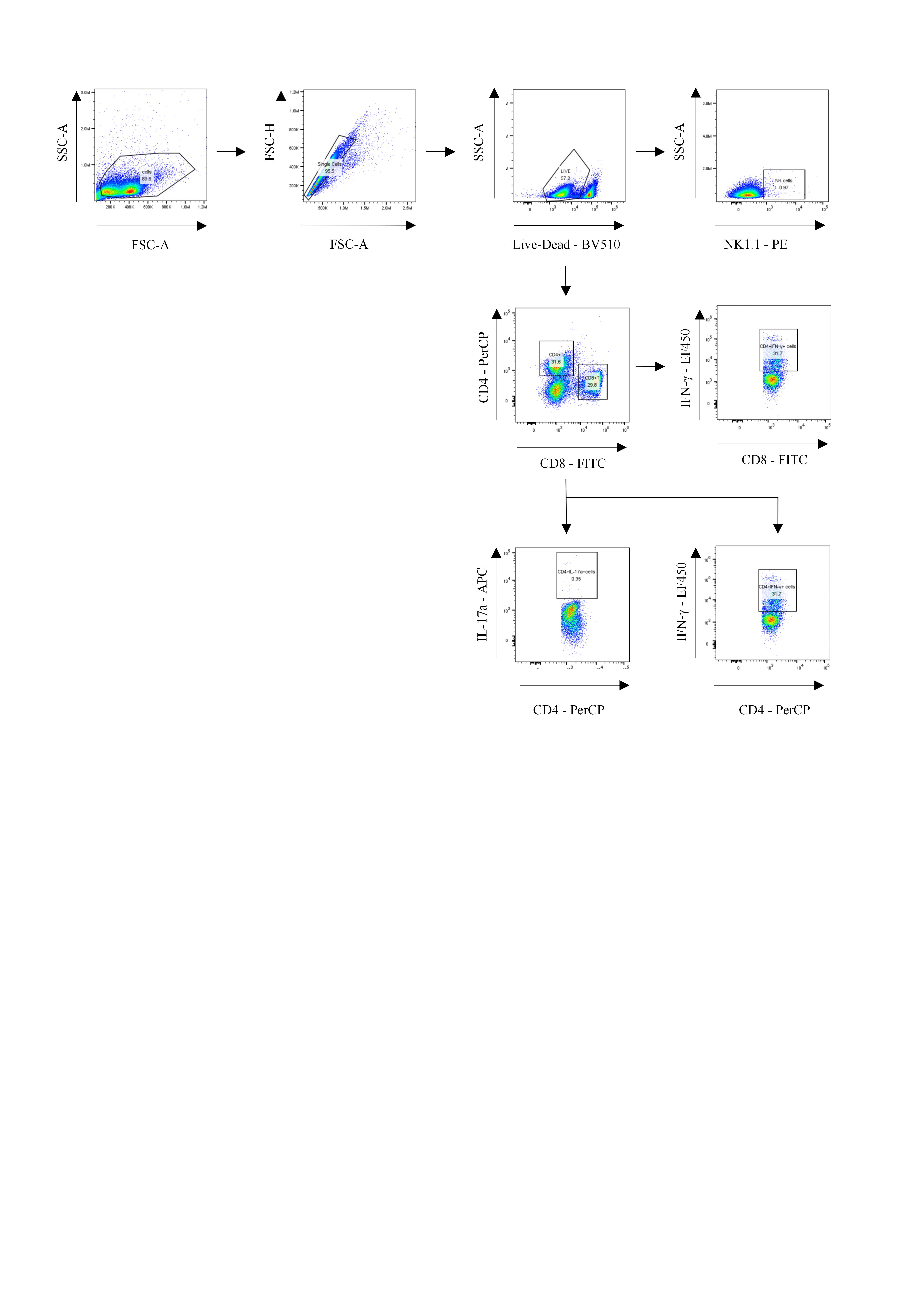

Supplement: Supplementary Figure 1 — Flow cytometry gating logic diagram showing the detection of cellular proportions in vitro and in vivo in this study. [file Image1.jpeg]

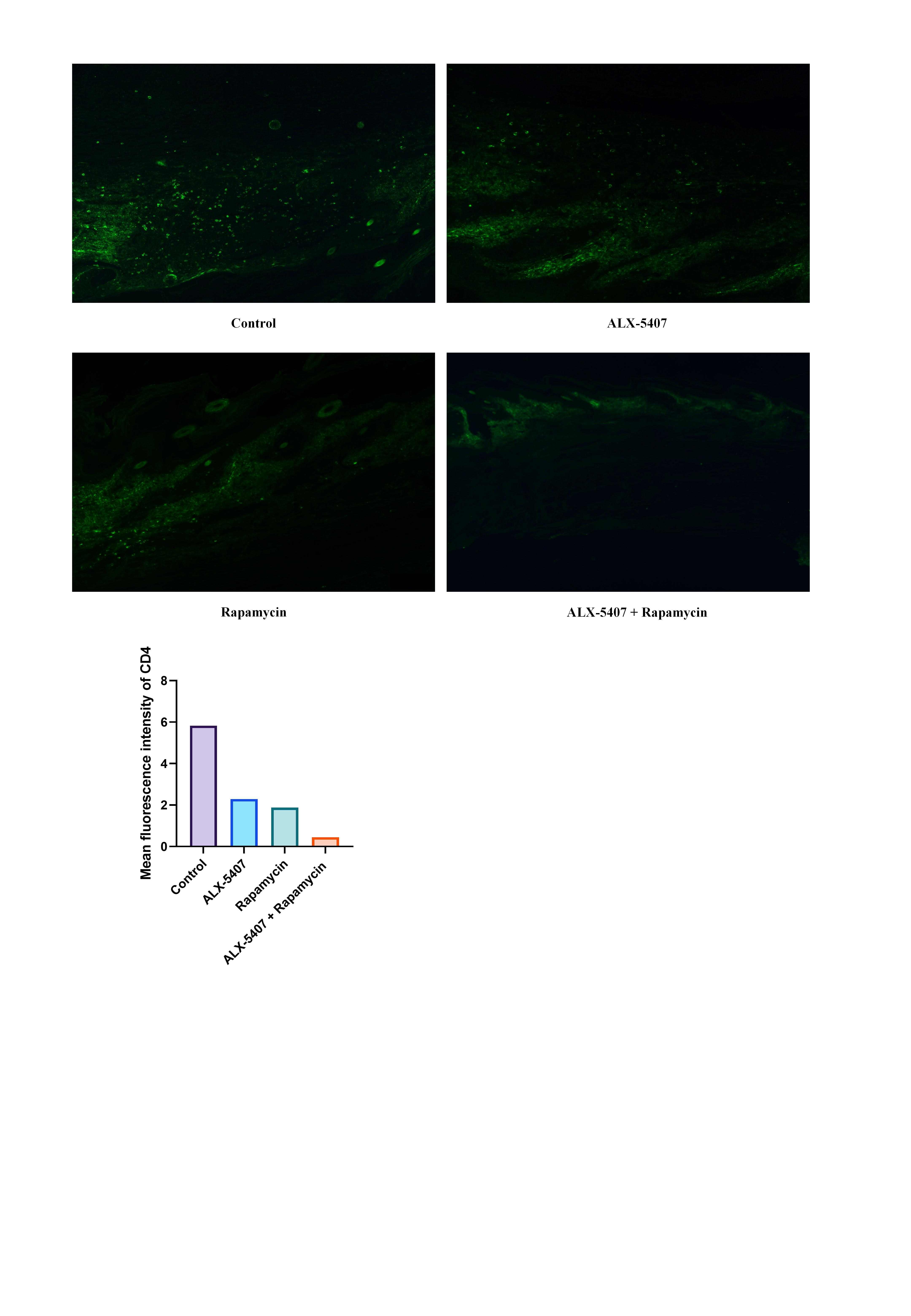

Supplement: Supplementary Figure 2 — Enlarged images of CD4 immunofluorescence staining in mouse transplanted skin graft sections in Figure 3D , and the comparison of CD4 fluorescence expression levels among different groups. [file Image2.jpeg]

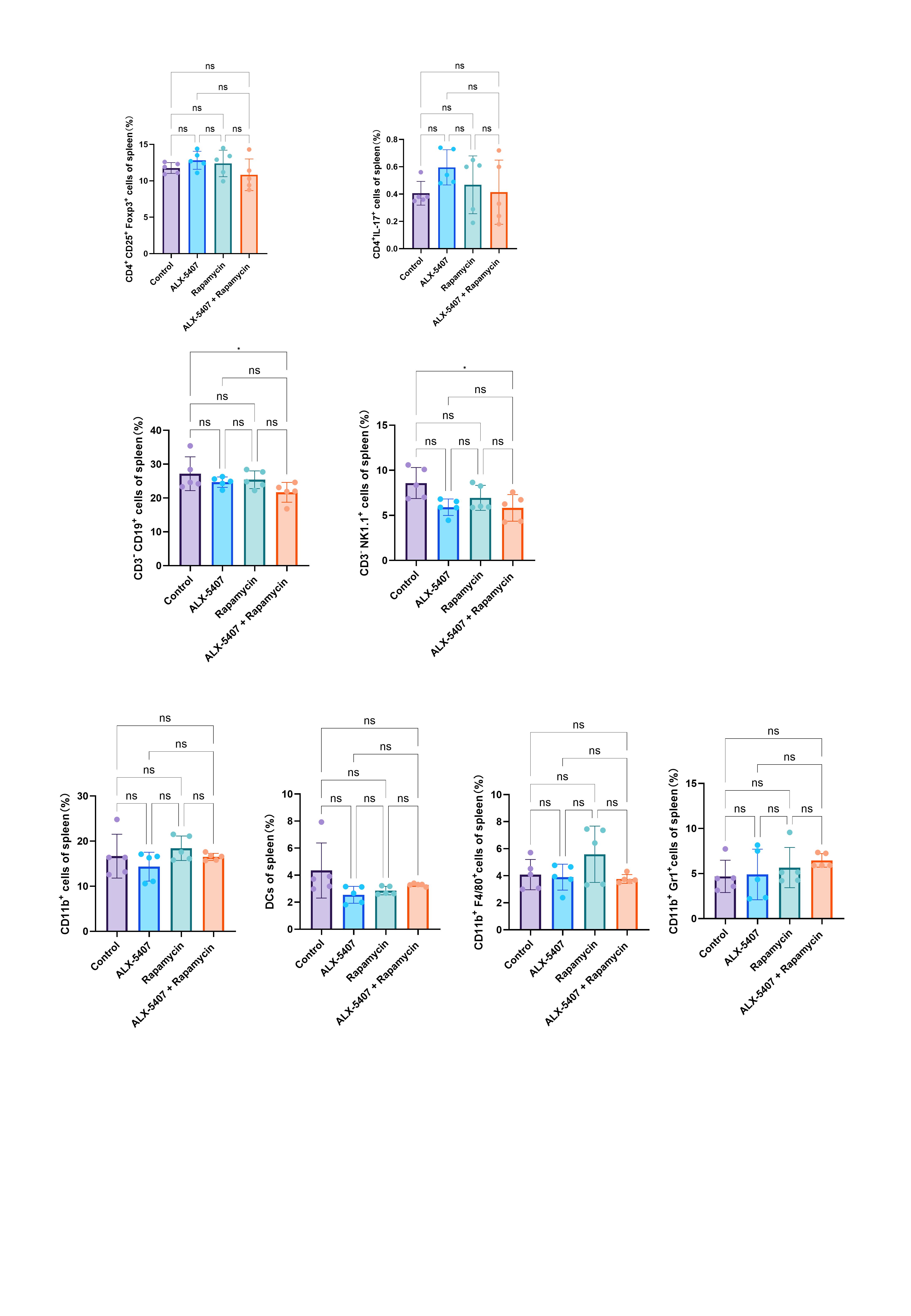

Supplement: Supplementary Figure 3 — ALX-5407 does not statistically significantly affect other cell populations in the spleen of skin-transplanted mice. BALB/C mouse skin was transplanted onto the backs of C57BL/6 mice, which were then randomly divided into four groups: control, ALX-5407, rapamycin, and ALX-5407 + rapamycin. Following the surgery, each group received daily intraperitoneal injections of their respective treatments. Seven days post-surgery, the mice were euthanized, and their spleens and peripheral blood were collected for flow cytometry analysis. The proportions of various cell populations across the groups were compared using ANOVA or T-tests. (n =5;*p < 0.05, ** p < 0.01, *** p < 0.001, **** p < 0.0001). [file Image3.jpeg]
